# Evaluation of Host Depletion and Extraction Methods for Shotgun Metagenomic Analysis of Bovine Vaginal Samples

**DOI:** 10.1128/spectrum.00412-21

**Published:** 2022-04-11

**Authors:** Chian Teng Ong, Gry Boe-Hansen, Elizabeth M. Ross, Patrick J. Blackall, Conny Turni, Ben J. Hayes, Ala E. Tabor

**Affiliations:** a Queensland Alliance for Agriculture and Food Innovation, Centre for Animal Science, The University of Queensland, Brisbane, Queensland, Australia; b School of Veterinary Science, Faculty of Science, The University of Queensland, Brisbane, Queensland, Australia; c School of Chemistry and Molecular Bioscience, Faculty of Science, The University of Queensland, Brisbane, Queensland, Australia; Huazhong University of Science and Technology

**Keywords:** cattle, metagenomics, microbiome, shotgun, vagina, veterinary microbiology

## Abstract

The reproductive tract metagenome plays a significant role in the various reproductive system functions, including reproductive cycles, health, and fertility. One of the major challenges in bovine vaginal metagenome studies is host DNA contamination, which limits the sequencing capacity for metagenomic content and reduces the accuracy of untargeted shotgun metagenomic profiling. This is the first study comparing the effectiveness of different host depletion and DNA extraction methods for bovine vaginal metagenomic samples. The host depletion methods evaluated were slow centrifugation (Soft-spin), NEBNext Microbiome DNA Enrichment kit (NEBNext), and propidium monoazide (PMA) treatment, while the extraction methods were DNeasy Blood and Tissue extraction (DNeasy) and QIAamp DNA Microbiome extraction (QIAamp). Soft-spin and QIAamp were the most effective host depletion method and extraction methods, respectively, in reducing the number of cattle genomic content in bovine vaginal samples. The reduced host-to-microbe ratio in the extracted DNA increased the sequencing depth for microbial reads in untargeted shotgun sequencing. Bovine vaginal samples extracted with QIAamp presented taxonomical profiles which closely resembled the mock microbial composition, especially for the recovery of Gram-positive bacteria. Additionally, samples extracted with QIAamp presented extensive functional profiles with deep coverage. Overall, a combination of Soft-spin and QIAamp provided the most robust representation of the vaginal microbial community in cattle while minimizing host DNA contamination.

**IMPORTANCE** In addition to the host tissue collected during the sampling process, bovine vaginal samples are saturated with large amounts of extracellular DNA and secreted proteins that are essential for physiological purposes, including the reproductive cycle and immune defense. Due to the high host-to-microbe genome ratio, which hampers the sequencing efficacy for metagenome samples and the recovery of the actual metagenomic profiles, bovine vaginal samples cannot benefit from the full potential of shotgun sequencing. This is the first investigation on the most effective host depletion and extraction methods for bovine vaginal metagenomic samples. This study demonstrated an effective combination of host depletion and extraction methods, which harvested higher percentages of 16S rRNA genes and microbial reads, which subsequently led to a taxonomical profile that resembled the actual community and a functional profile with deeper coverage. A representative metagenomic profile is essential for investigating the role of the bovine vaginal metagenome for both reproductive function and susceptibility to infections.

## INTRODUCTION

The term metagenome refers to the genomes and genes of the microorganisms present in a defined environment (1). Metagenomic studies can bring forth a breakthrough to microbial discovery and ecology studies. For instance, the discovery of nonculturable and new microbial species has been challenging with traditional culture-based and molecular approaches and is now commonly reported by various metagenomic sequencing projects (2, 3). The entire microbial community can be accessed without extensive labor and a stringent need to customize a suitable growth environment or specific markers for each microbial species (4, 5). Profiling the microbial community in host systems has revealed a greater coverage of microbial inhabitation in host systems, including those which were long presumed to be sterile, for example, urine (6), milk (7), fetal fluid (8), and uterus (9). The microbiome was demonstrated to play a vital role in host physiology (10, 11), nutrition (12, 13), and health (14, 15). A balanced microbiome is beneficial to healthy biological processes such as digestion, immune system maturation, toxin degradation, and pathogen defense (16). However, imbalanced microbiomes have been associated with disease development, such as urinary tract infection (17) and reproductive diseases (18). In addition to the interrogation of the microbial community profile, diversity, and abundances, the untargeted shotgun metagenomic approach also provides insights into the functional characteristics of the microbiome in the complex host-microbial relationships under different circumstances (19–21).

Increasing research efforts have been directed to bovine reproductive tract metagenomic studies in the past 10 years. Metagenomes of bovine reproductive tracts have been associated with bovine reproductive functions, including reproductive cycle (22), gestation (23), and reproduction health (24, 25). Microbial infiltration may occur during calving or mating and lead to undesirable shifts of the metagenome in the bovine reproductive tract (26–28). Disturbed commensal microflora have been associated with the development of reproductive diseases, including metritis and endometritis (29, 30).

One of the challenges in host-derived metagenomic studies is host DNA contamination, which has been commonly observed in most host-derived microbiome studies (31–34). In addition to the vaginal epithelial tissue collected during the sampling process, metagenome samples from the bovine reproductive tract are saturated with large amounts of extracellular DNA and secreted proteins essential for various physiological purposes, including reproductive cycle and host defense (35, 36). The vast amount of host genetic content in the metagenome sample dominates the sequencing capacity, and, in turn, reduces the sequencing depth for the metagenomic content (37). Additionally, the host genome is larger than the average microbial genome. Hence, host genomic information could easily dominate the sequencing capacity even with a small number of host cells (38). For instance, the cattle genome is 2.7 Gb, approximately 750 times larger than the average bacterial genome (∼3.6 Mb) (39, 40). Importantly, the ratio of host-to-microbe cells varies at different sampling sites. For example, fecal samples typically yield much less host genomic material than saliva, mucus, skin, and vaginal swabs. Additionally, metagenome samples constitute a mixture of microorganisms, including bacteria, fungi, archaea, viruses, and protists, which represent different cell structures. It is challenging to efficiently extract a mixture of microbial DNA using one extraction method (37, 38). While increasing sequencing depth can recover representative metagenomic profiles from metagenome samples with a large amount of host genomic materials (34), this increases the costs and requires extensive computational effort. Therefore, host depletion is pivotal for cost-effective metagenomic studies of host-derived metagenome samples.

In most of the bovine vaginal metagenomic studies, DNeasy Blood and Tissue extraction kit (DNeasy) was incorporated for metagenome DNA extraction (41). However, the benefits of effective host depletion for metagenomic studies were reported, primarily for human clinical samples (42–45). In general, these methods manipulate the differences between mammalian cells and microbial cells, for example, cell size, cell wall structures, and DNA methylation pattern, to achieve the separation either before or after DNA extraction. Nonetheless, the different complexity and chemistry matrix of the host-derived samples pose different degrees of impact on the performances of the host depletion and extraction methods (38, 46). Therefore, we conducted the first study to evaluate the efficacies and potential bias of the different host depletion and extraction methods on bovine vaginal samples intended for metagenomic studies. In total, three host depletion methods and two extraction methods were evaluated from different aspects, including the percentage of 16S ribosomal (r)RNA genes in the extracted samples, the percentage of microbial reads, the coverage and accuracy of the metagenomic profiles.

## RESULTS

### Spiked suspension.

The total amount of spiked bacteria in each of the vaginal swabs, 14 mock samples, and 14 positive-control samples was 2.3 × 10^7^ CFU/mL (Table 1). According to culture plate counting, Pseudomonas aeruginosa constituted the highest percentage (43.01%), followed by Campylobacter fetus subsp. venerealis (19.73%) and Aliarcobacter cryaerophilus (19.73%), Enterococcus. faecalis (9.63%), and Staphylococcus aureus (7.89%).

**TABLE 1:** Details of the bacteria used to assess the effectiveness of the depletion and DNA extraction methods

Bacterial species	Strain	Gram stain	Aerotolerance	Median GC (%)	Genome size (Mb)	Spiked concentration (CFU/mL × 10^6^)
Aliarcobacter cryaerophilus	CCUG17804	Gram-negative	Aerobic	27.4	2.09	4.55
Campylobacter fetus subsp. venerealis	ATCC 19438	Gram-negative	Microaerophilic	33.1	1.82	4.55
Enterococcus faecalis	BR1200	Gram-positive	Facultative anaerobe	37.4	2.97	2.22
Pseudomonas aeruginosa	ATCC 27853	Gram-negative	Aerobic	66.2	6.6	9.91
Staphylococcus aureus	ATCC 29213	Gram-positive	Anaerobic	32.7	2.84	1.82

### Sample preparation and extraction.

**FIG 1:**
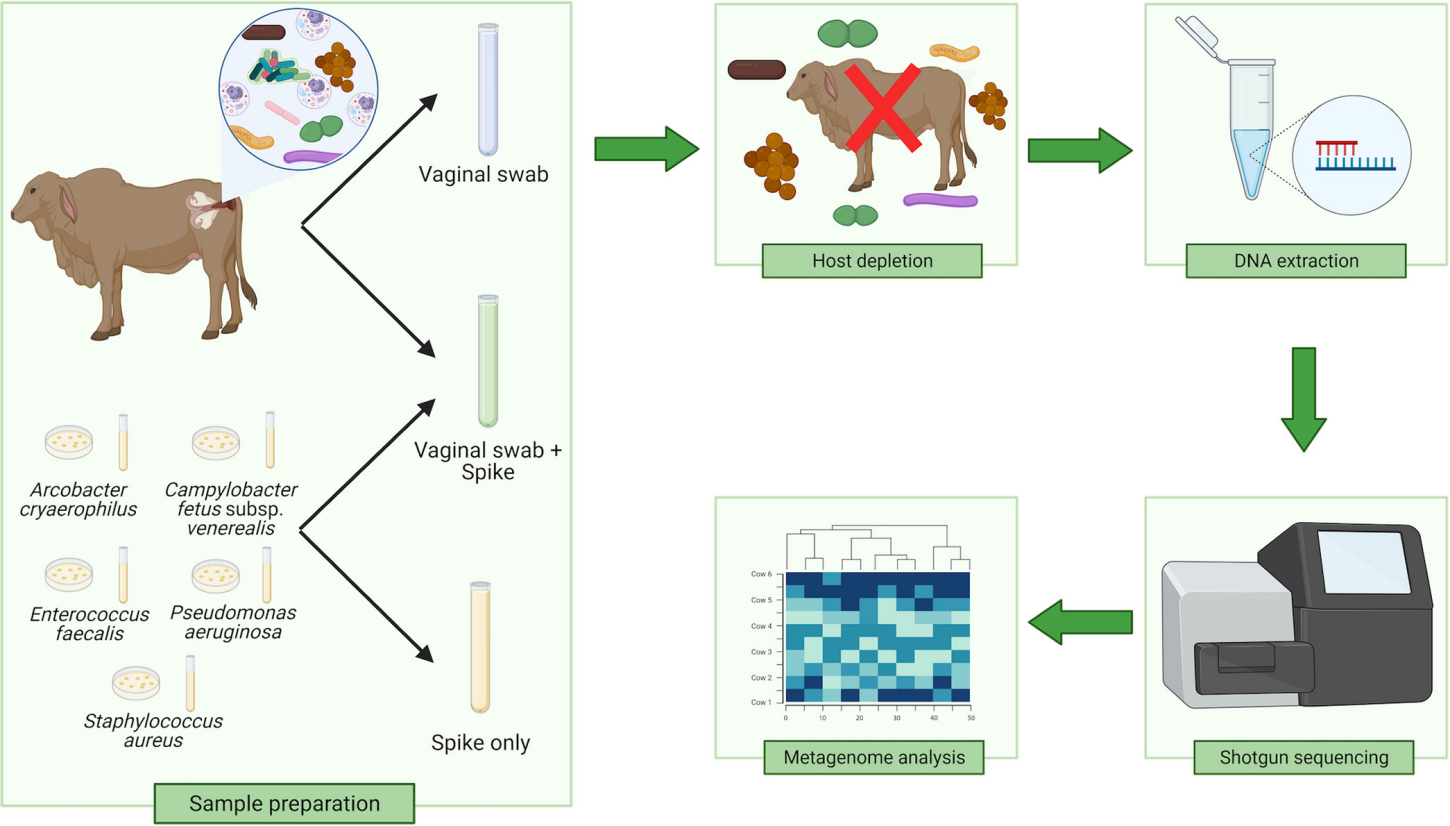
Experimental design. Three sample types were used to examine the efficacies of depletion and extraction methods. Vaginal swabs were vaginal swabs without spiked bacteria. The mock sample contained vaginal samples and five bacterial suspensions. Positive-control samples contained only the five bacterial suspensions. The samples were treated with different depletion and extraction methods to obtain the nucleic acids for shotgun sequencing and subsequent metagenomic analysis.

In total, 42 samples were prepared, including 14 vaginal swabs, 14 mock samples, and 14 positive-control samples (Fig. 1).

### Shotgun sequencing.

Samples processed using the combination of NEBNext and QIAamp extraction were eliminated from downstream analyses because the DNA quantities were too low and were not eligible for shotgun sequencing. Whole-metagenome shotgun sequencing of the samples (*n* = 36) returned an average of 6.47 Gbp of data and an average of 51,956,238 raw paired-end reads per sample. An average of 48,965,875 paired-end reads per sample remained after trimming (Text S1).

### Metagenome assembly.

The paired-end reads were assembled into contigs, and the accuracy of contig-based metagenomic profiles was investigated. The number of contigs ranged from 335 to 19,711 and the average contig length ranged from 484 to 48,911 (Text S1). The contig coverage was examined by realigning the reads back to the contigs, and the mapped percentage ranged from 15.03 to 99.80% (Text S1). The mapped percentage of the assembled contigs evaluated using MetaQUAST ranged from 24.06 to 99.88% (Text S1).

### Relationship between the percentage of 16S rRNA genes and the percentage of microbial reads in the samples.

**FIG 2:**
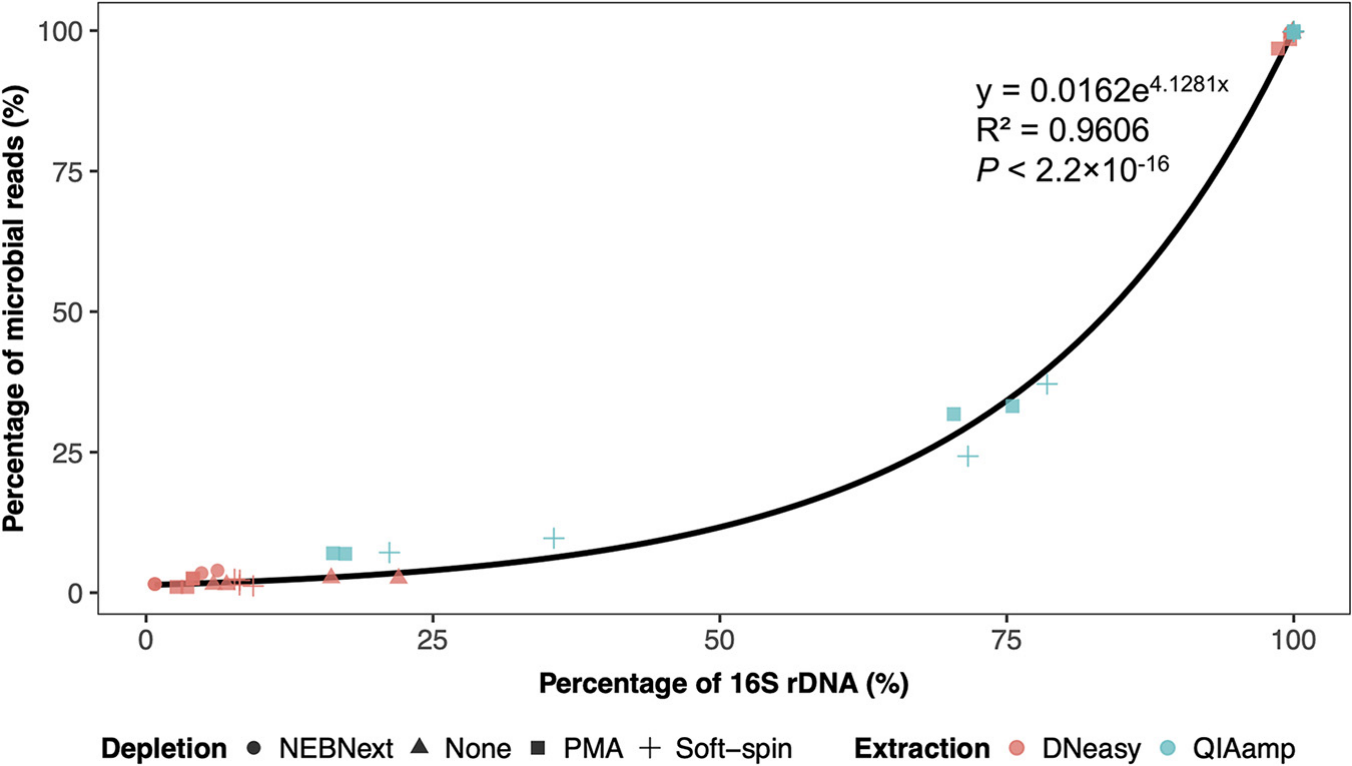
The relationship between the percentage of 16S rRNA genes and the percentage of microbial reads identified in the bovine vaginal samples. The markers represent all the samples involved in this study (*n* = 36), including vaginal swabs, mock samples, and positive-controls. Samples were treated with different depletion methods, including None (circle), NEBNext (triangle), PMA (square), and Soft-spin (cross). Red represents samples extracted using DNeasy and blue represents samples extracted using QIAamp.

To analyze the impacts of host DNA depletion in the bovine vaginal samples, the relationship between the percentage of the 16S rRNA genes and the percentage of microbial reads identified in the samples (*n* =36) was investigated (Fig. 2). The result indicated the proportion of microbial reads sequenced by shotgun sequencing was exponentially proportional (*P* < 0.001) to the percentage of 16S rRNA genes in the bovine vaginal samples. The impacts of depletion and extraction methods on the proportion of 16S rRNA genes and microbial reads were not significantly different. However, Soft-spin and QIAamp recovered the highest percentages of 16S rRNA genes (mean = 53.3% and 65.5%) and microbial reads (mean = 40.4% and 46.4%), respectively.

### Impacts on the taxonomic profile.

**FIG 3:**
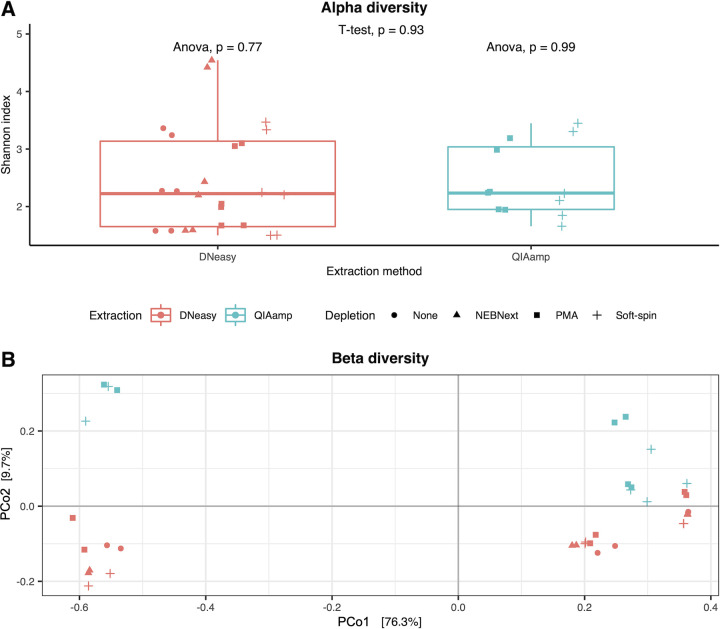
Alpha (A) and Beta (B) diversity of the samples involved in this study (*n* = 36), including vaginal swab, mock sample, and positive-control. Samples were treated with different depletion methods, including None (circle), NEBNext (triangle), PMA (square), and Soft-spin (cross). Red represents samples extracted using DNeasy and blue represents samples extracted using QIAamp. ANOVA test was applied to examine the significance of differences between the alpha diversity of samples treated with different depletion methods while the *t* test was applied to examine the significance of differences between the alpha diversity of samples processed with different extraction methods. Beta diversity was represented by principal coordinate analysis ordination of the Bray-Curtis dissimilarity matrix.

Alpha diversity of each sample (*n* = 36) was examined using the Shannon index. In general, there was a higher alpha diversity in the vaginal swab (mean = 3.45) followed by a mock sample (mean = 2.21) and positive-control (mean = 1.67). However, the alpha diversities between samples treated with different depletion methods (ANOVA, *P* = 0.77 and 0.99) and different extraction methods (*t* test, *P* = 0.93) were not significantly different (Fig. 3A). The Bray-Curtis dissimilarity demonstrated that the dissimilarity caused by depletion methods was not significant (permutational multivariate analysis of variance [PERMANOVA], *P* = 0.58) while the dissimilarity resulting from the extraction methods was significant (PERMANOVA, *P* = 0.005) (Fig. 3B).

**FIG 4:**
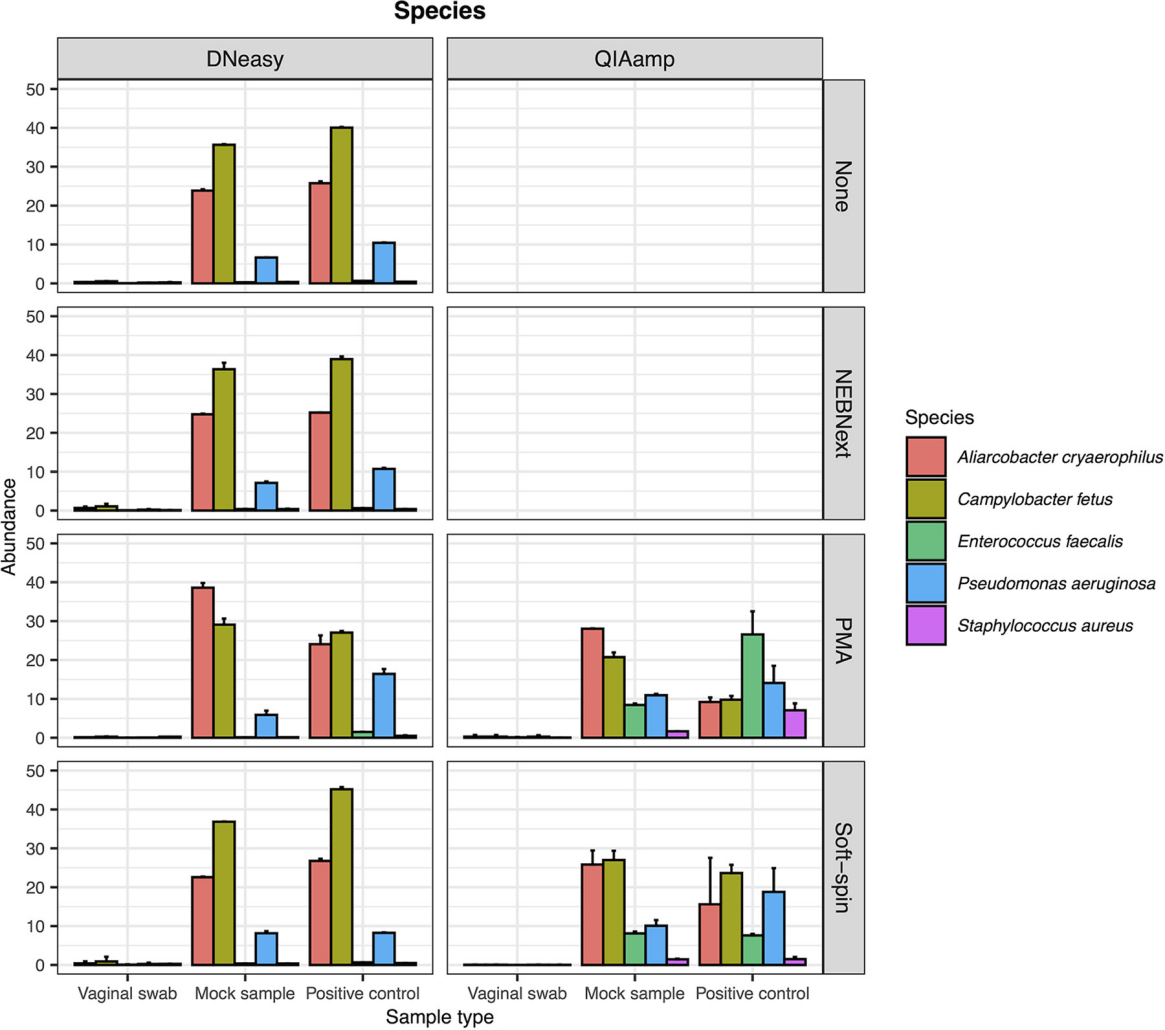
Abundances of spiked bacteria, including Aliarcobacter cryaerophilus, Campylobacter fetus, Enterococcus faecalis, Pseudomonas aeruginosa, and Staphylococcus aureus, in the samples involved in this study (*n* = 36), including vaginal swabs, mock samples, and positive-controls.

The abundances of the bacteria spiked into the mock sample and positive-control were examined to assess the impacts of depletion and extraction methods on the recovery of taxonomic profile. The abundances of the spiked bacteria in the negative controls (vaginal swab) samples were less than 2% (Fig. 4). The impacts of depletion methods on the abundances of the spiked bacteria were not significantly different from each other (Table 2). Contrarily, the impacts of the extraction method were insignificant on the abundances of A. cryaerophilus, C. fetus, and P. aeruginosa but were significant for E. faecalis (ANOVA, *P* < 0.001) and S. aureus (ANOVA, *P* = 0.003). Enterococcus faecalis and S. aureus were detected at lower abundances (mean = 0.404% and 0.34%) in samples treated with DNeasy compared to QIAamp (mean = 8.47% and 1.96%). Enterococcus faecalis and S. aureus constituted 9.63% and 7.89% in the spiked suspension (Table 1). Hence, QIAamp was demonstrated to recover percentages of bacteria that were closer to the spiked proportion.

**TABLE 2:** Significance of different, calculated using ANOVA test, between the abundances of spiked bacteria in the samples (*n* = 36) processed with different depletion and extraction methods

	ANOVA, *P* value	
Species	Depletion	Extraction
Aliarcobacter cryaerophilus	0.991	0.32
Campylobacter fetus	0.444	0.065
Enterococcus faecalis	0.195	0.000156
Pseudomonas aeruginosa	0.861	0.189
Staphylococcus aureus	0.27	0.003

### Impacts on functional classification.

**FIG 5:**
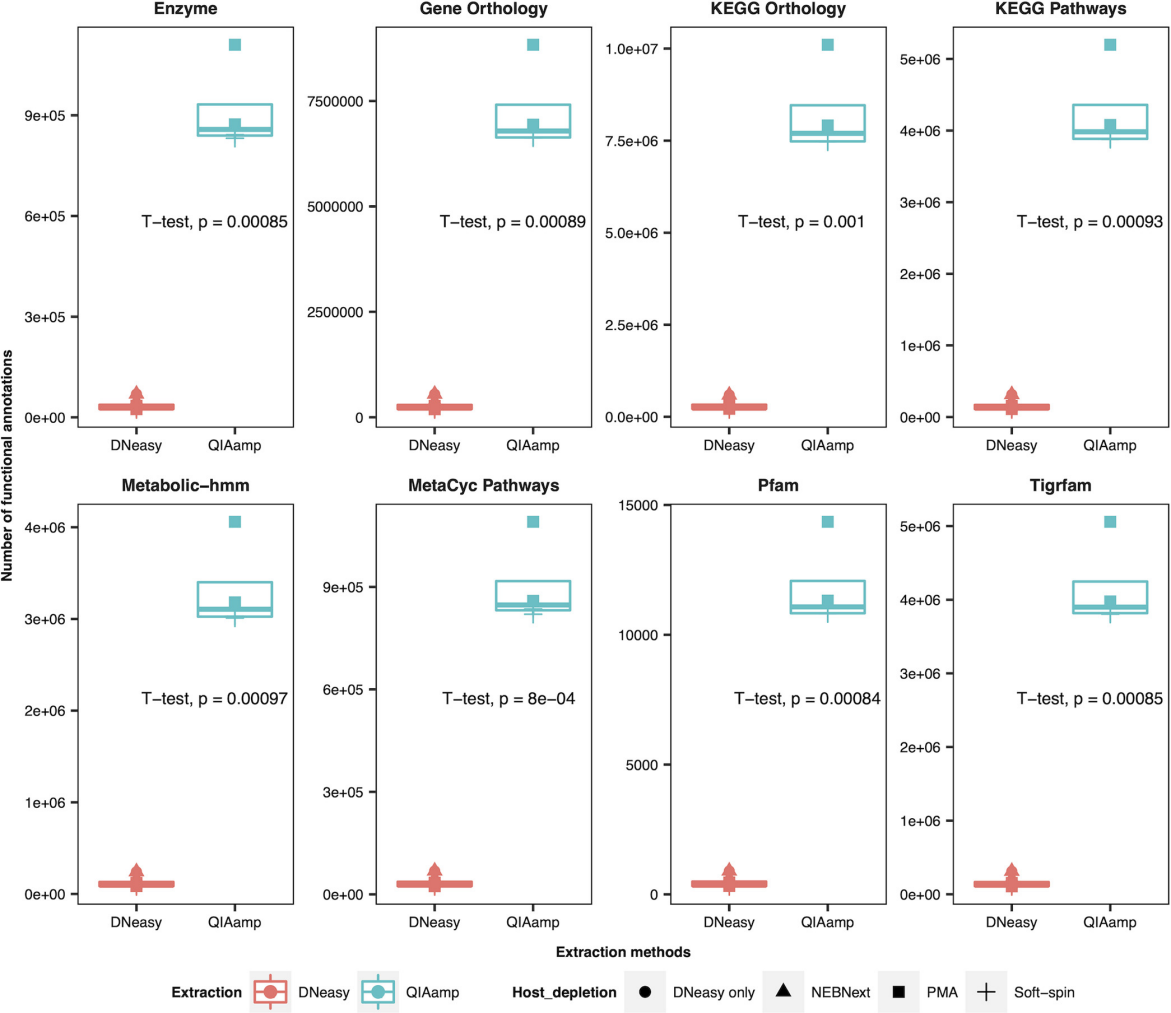
Number of functional annotations identified by the different functional and pathway databases. A *t* test was applied to examine the significance of differences between the number of functional annotations in samples processed with different extraction methods.

To study the impacts of host depletion and extraction methods on the contig-based functional classification of shotgun metagenomic data, the total functional annotations were reported by eight databases, including Enzyme, Gene Orthology, KEGG Orthology, KEGG pathways, Metabolic-hmm, MetaCyc pathways, Pfam, and Tigrfam. The results indicated that the extraction methods contributed significant impacts on the number of functional annotations reported (*P* < 0.001) (Fig. 5).

## DISCUSSION

This is the first study that has evaluated multiple host depletion and metagenome extraction methods on bovine vaginal metagenome samples. The exponential relationship between the percentage of 16S rRNA genes and microbial reads depicted in this study suggested the importance of host depletion for efficient metagenome sequencing. Host DNA contamination reduced the sequencing depth for metagenome content and subsequently led to reduced sensitivity of metagenomic studies performed with untargeted shotgun sequencing (47). The results indicated that Soft-spin and QIAamp outperformed the other host depletion and extraction methods, including the commonly used extraction method “DNeasy only,” in removing the cattle DNA from bovine vaginal samples. Consequently, samples extracted with QIAamp resulted in taxonomic profiles that more resembled the spiked community and functional profiles with deeper coverage.

The efficacy of PMA treatment was dependent on the microbial content in the metagenome samples. Propidium monoazide unbiasedly digests the exposed DNA. Hence, the selective impacts of PMA-based host depletion treatments depend greatly on the lysis methods and the permeability of microbial cell membrane (38, 48, 49). Potentially, the integrity of the microbial cells in vaginal swab samples was not preserved in vaginal mucus during the sample collection and transfer. Hence, the exposed microbial DNA content was digested together with the cattle genomic materials before extraction. Metagenome samples treated with NEBNext recovered fewer 16S rRNA genes and microbial sequences than samples extracted without a host depletion step, indicating the low efficacy of NEBNext in depleting cattle genomic materials from vaginal samples. This is potentially due to the low molecular weight genomic DNA extracted using DNeasy and QIAamp. NEBNext is a postextraction method that requires >15 kb high-molecular-weight DNA, which is typically achieved by conventional extraction methods for effective capture and subsequent host removal (37). The low throughput of conventional extraction methods renders them less ideal for quantitative metagenomic studies with multiple samples (50, 51). Extraction methods used in this study were all based on the vigorous lysis required for bacterial cells, which is likely to shear the long fragment DNA in most cases, making the NEBNext protocol unsuitable for this use. Of the tested host depletion methods (Soft-spin, PMA treatment, and NEBNext), Soft-spin was the most efficient and consistent at depleting the cattle DNA from the bovine vaginal samples. It was demonstrated that Soft-spin resulted in higher percentages of 16S rRNA genes and microbial sequences. Soft-spin effectively separated the cattle tissues in the bovine vaginal samples and allowed a higher percentage of microbial DNA to be extracted.

DNeasy has been commonly incorporated in bovine reproductive tract metagenome studies (52–57), while QIAamp is relatively less common (29). However, our results indicated that QIAamp was more effective than DNeasy in maximizing the 16S rRNA genes and microbial sequences in both the actual and mock bovine vaginal samples. Unlike DNeasy, which relied solely on chemical lysis for nucleic acid extraction, QIAamp implemented two separate lysis steps. The first step was the host enzymatic lysis and degradation with benzonase. The efficacy of benzonase in nucleic acid hydrolyzation is well documented and has been widely used in biopharmaceutical production (46, 58, 59). The second step was the bead-beating mechanical lysis and proteinase K lysis of microbial cell walls. Therefore, metagenome samples extracted with QIAamp extraction recovered a higher percentage of microbial DNA and sequences, particularly E. faecalis and S. aureus, which were significantly less captured by the DNeasy extraction. Gram-positive bacteria like E. faecalis and S. aureus possess a thick outer cell wall made up of peptidoglycan, allowing Gram-positive bacteria to be more resistant to heat and chemical stresses (60, 61). Bead-beading mechanical lysis helps to break the thick microbial cell walls and release the encapsulated nucleic acid, consequently improving the yield and quality of microbial DNA (62, 63). The effectiveness of QIAamp in increasing the microbial DNA and the subsequent effects on the metagenomic profiles were also reported in other host-derived metagenomic studies, which investigated fluid collected from infected respiratory tract and biopsy specimen samples from diabetic foot infections (64, 65).

The vaginal samples used in this study were collected from acyclic heifers, which were often the control group for comparative bovine reproductive tract metagenomic studies (66–69). The mock sample simulated the bovine vaginal samples collected from mated cows or cows that develop reproductive diseases, which often have richer microbial content (70). Vaginal swabs are one of the most challenging sample types for metagenomic investigation due to the high (>90%) host DNA content (38, 47). Our findings demonstrated that Soft-spin and QIAamp were effective in extracting the metagenomic content from bovine vaginal samples, either with low or enriched microbial contents and subsequently increased the sequencing efficiency and recovered metagenome profiles with higher accuracy and coverage. This study provides a beneficial reference for other host-derived metagenomic studies, especially samples with a similar chemistry matrix and low microbial content as bovine vaginal swabs.

One of the greatest advantages of sequencing the metagenome samples with untargeted shotgun methods is the functional profiling of the metagenome. The assembled contigs can be mapped to the protein and pathway databases for functional annotations to investigate the functional focus of the metagenome under different conditions (21, 71). Our results demonstrated that there was a significantly higher recovery rate of the functional annotations in the samples extracted using QIAamp. The increased number of functional annotations identified, and the relative coverage of each annotation essentially improved the functional profiles of metagenome samples by QIAamp. An extensive functional profile with deep coverage provides information regarding the metagenome's functional capacity, including the virulence factors, antibiotic resistance, and metabolic pathways (72). Nonetheless, the accuracy of the functional profile identified by QIAamp was not explored in this study.

Because samples in this study were collected from healthy heifers, the impacts of infected host tissues and the influx of inflammatory host cells on the extracted metagenomic content remain unexplored. Further studies shall investigate the host-to-microbe ratio in samples collected from cattle diagnosed with reproductive diseases and its impacts on the sequencing efficiency and recovery of metagenomic profiles. There were only 2 replicates used in the method tested for each sample, this potentially undermined the credibility of the result. Additionally, the proportion of each spiked bacteria in the extracted DNA shall be verified using an individual quantification test.

Our results demonstrated that Soft-spin was more efficient than NEBNext and PMA in reducing the host-to-microbe ratio in bovine reproductive tract metagenome samples. The relationship between 16S rRNA genes and microbial reads detected signified that the shotgun metagenomic sequencing was increasingly more efficient as the percentage of 16S rRNA genes in the metagenome samples increased. QIAamp extraction outperformed DNeasy by improving the microbe-to-host DNA ratio, providing more accurate taxonomic profiles, and increasing the sensitivity in deciphering the functional profiles of bovine reproductive tract metagenome samples. This study provides optimal metagenomic conditions for the application in future bovine vaginal reproductive microbiome research.

## MATERIALS AND METHODS

### Sample collection and transportation.

Samples were collected from 30 Droughtmaster heifers from a cattle farm in Northern Queensland under UQ Animal Ethics Approval AE30009. An experienced veterinarian conducted cattle health assessments and sample collection. The heifers were between 12 and 18 months old during sample collection. Using transrectal ultrasound, the heifers were defined as not pregnant and without the presence of a corpus luteum, but with the presence of either small or large follicles. The heifers were defined as being acyclic, likely in the late prepubertal period. The heifers' average live weight, hip height, and body score were 316.5 ± 30.7 kg, 193 ± 43 cm, and 2.8 ± 0.25 (scale 0 to 5), respectively. Vaginal samples from the heifers were collected using the Tricamper (DAF Queensland, Australia) sampling tool following the manufacturer’s protocol. Briefly, the Tricamper was inserted into the cow’s vagina with the leading edge in contact with the dorsal wall of the vagina. The Tricamper was moved back and forth in the vagina to collect the swab. Upon removing the Tricamper from the vagina, the other end of the Tricamper was blocked to prevent spillage. The swab sample was immediately preserved in a 10 mL tube preloaded with 5 mL phosphate-buffered saline (PBS) by excising the head of the Tricamper device. The samples were kept on ice during delivery and were processed within 6 h upon arrival to the laboratory. Each sample was first vortexed for 15 s and followed by an additional 15 s of vortex after the Tricamper head was removed from the tube. The vaginal mucus samples were then transferred into a new sterile tube.

### Spiked bacteria and mock bacterial community.

To monitor the recovery of the metagenomic profiles, five bacteria were used for spiking, Pseudomonas aeruginosa (ATCC 27853), Staphylococcus aureus (ATCC 29213), Campylobacter fetus subsp. *venerealis* (ATCC 19438), Aliarcobacter previously Arcobacter cryaerophilus (312), and Enterococcus faecalis (BR1200). Aliarcobacter cryaerophilus and C. fetus subsp. venerealis are pathogens associated with abortion and infertility in cattle (73, 74). Enterococcus faecalis, P. aeruginosa, and S. aureus are commonly isolated from bovine reproductive tract samples but are regarded as normal flora or environmental organisms (75–77). Additionally, these bacteria formed a range of characteristics in terms of genome sizes, Gram stain, GC content, and aerotolerance, which were hypothesized to affect the efficiencies of host depletion methods and DNA extraction methods differently. Aliarcobacter cryaerophilus, E. faecalis, P. aeruginosa, and S. aureus were cultured on the BBL Brucella agar (Becton, Dickinson) under aerobic conditions at 37°C for 24 h. Campylobacter fetus subsp. venerealis were cultured on the tryptone soya agar (TSA) supplemented with 5% defibrinated sheep blood (Oxoid) under microaerophilic conditions at 37°C for 48 h. Colonies of each bacterial isolate were resuspended in sterile PBS to reach an optical density measured at wavelength 600 nm (OD_600_) for yielding colony count of approximately 1 × 10^8^ CFU/mL.

### The mock bacterial community in heifer vaginal swab samples.

The 30 vaginal swab samples were combined and homogenized in a sterile bottle. Five milliliters of the homogenized vaginal swab samples were then redistributed into 28 new sterile tubes. The vaginal swab with no spiked bacteria was labeled as “vaginal swab”. The “mock samples” were prepared by adding 0.1 mL of each bacterial suspension into 5 mL homogenized vaginal swab samples. “Positive controls” were prepared by adding 0.1 mL of each bacterial suspension to 5 mL of sterile water. All samples were prepared in duplicates.

The bacterial load was monitored by the plate count method. Serial dilutions were made from the bacterial suspensions, and 0.1 mL of each dilution was plated onto TSA supplemented with 5% defibrinated sheep blood. For P. aeruginosa, S. aureus, and E. faecalis, the number of colonies was counted after 24 h. For A. cryaerophilus and C. fetus subsp. venerealis, the number of colonies was counted after 48 h.

### Host depletion and extraction methods.

Two of each sample type, including vaginal swabs, mock samples, and positive-controls, were processed with one of the depletion methods, including “None,” “NEBNext,” “Soft-spin” and “PMA”. After being treated with the depletion method, the samples were extracted with either “DNeasy” or “QIAamp”.

### Slow and short centrifugation (Soft-spin).

Slow and short centrifugation (Soft-spin) was performed for respective samples before extraction. The samples were centrifuged at 1000 × *g* for 1 min at 4°C. Without disturbing the pellet, the supernatant was collected into a new tube for DNA extraction.

### NEBNext Microbiome DNA Enrichment kit (NEBNext).

NEBNext is a postextraction host depletion method. Extracted DNA was processed with the NEBNext Microbiome DNA Enrichment kit (NEBNext) according to the manufacturer’s protocol. Briefly, one microgram of the extracted DNA was mixed with magnetic beads bound to the methyl-CpG binding domain (MBD) to capture the methylated host DNA. A magnetic rack was used to separate the captured host DNA. The supernatant, which contained the microbial DNA was precipitated and diluted in TE buffer (10 mM Tris/0.1 mM EDTA, pH 8).

### Osmosis and propidium monoazide treatment (PMA).

PMA is a preextraction host depletion method. Samples were processed according to a published protocol (38). Briefly, vaginal samples were centrifuged at 4000 × *g* for 10 min to obtain the cell pellet. The pellet was resuspended in 0.2 mL of sterile water by pipetting and brief vortexing. The suspension was left at room temperature for 5 min to allow mammalian cell lysis by osmosis. A 10 μL aliquot of 0.2 mM PMA dye (Biotium) solution was added to each of the samples. The mixtures were briefly vortexed to ensure thorough mixing and were then incubated in the dark for 5 min at room temperature. Subsequently, the samples were placed on ice within 25 cm of a light source for 25 min to allow light activation of the PMA molecule. The samples were briefly vortexed at 5-min intervals with light exposure.

### Qiagen DNeasy blood and tissue kit (DNeasy).

The sample was centrifuged at 4000 × *g* for 15 min to obtain a cell pellet. The supernatant was discarded, and DNA was extracted from the cell pellet according to the manufacturer’s instructions for Gram-positive bacteria. Briefly, the pellet was treated with enzymatic lysis buffer and proteinase K digestion before column precipitation. Precipitated DNA was washed and eluted in 60 μL of TE buffer.

### Qiagen QIAamp DNA Microbiome kit (QIAamp).

DNA was extracted according to the manufacturer’s instructions. First, the sample was centrifuged at 4000 × *g* for 15 min to obtain the cell pellet. Briefly, the pellet was subjected to lysis buffer, benzonase enzyme, bead beating, and pathogen lysis buffer before column precipitation. The precipitated DNA was eluted in 60 μL of TE buffer.

### Quantitative PCR (qPCR) assay and cycling conditions.

To quantify the bacterial copy number, qPCR was performed on the extracted DNA with PowerUp SYBR Green Master Mix (Applied Biosystems) using 16S rRNA gene-specific primers, 764F and 907R, for bacteria (78). Primers encompassing the beta2-microglobulin (B2M) gene were utilized for estimating the cattle copy number (79) (Table 3). Thermal cycling was initiated with the activation of Uracil-DNA Glycosylase at 50°C for 2 min followed by incubation of the DNA polymerase at 95°C for 2 min and 40 cycles of denaturing and annealing steps, which were 95°C for 15 s and primer-pair specific annealing temperature for 15 s, respectively. The qPCR was finished with an extension at 72°C for 1 min. Serial dilutions of cattle blood DNA and cultured bacterial samples were amplified alongside to generate the standard curves. All qPCR assays, including the nontemplate controls, were performed in duplicates. Results were generated using a CFX96 real-time PCR detection system (Bio-Rad) and data were analyzed using CFX Maestro software (Bio-Rad) with the Cq thresholds determined by the software.

**TABLE 3:** List of primers used in quantitative PCR for bacterial and cattle DNA quantification

Target gene	Primer ID	Primer sequence	Amplicon size	Annealing temp	Reference
B2M	B2M-F	ACCTGAACTGCTATGTGTATGG	134 bp	58°C	(79)
	BRM-R	GTGGGACAGCAGGTAGAAAG			
16S rRNA	764-F	CAAACAGGATTAGATACCC	143 bp	54°C	(110, 111)

The amount of amplicon was calculated from the standard curves generated with known concentrations of 16S rRNA genes and cattle blood DNA. The estimated copy number of cattle and bacteria was calculated as previously described (80). Briefly, the amount of amplicon (SQ) was calculated from the standard curves generated with known concentrations of bacterial DNA and cattle blood DNA. The estimated copy number of cattle and bacteria were derived from the ratio of SQ in gram multiplied by the Avogadro's number N_A_ (6.0221 × 10^23^ mol^−1^) and length of amplicon multiplied by the mean molar mass of a base pair M_Bp_ (660 g mol^−1^). The estimated copy number of cattle and bacteria were determined to compute the percentage of 16S rRNA genes.

### Shotgun sequencing, quality filtering, and contamination read removal.

The quantity of extracted DNA was measured using Qubit 4 fluorometer (Invitrogen) with either Qubit dsDNA Broad Range assay kit (Invitrogen) or Qubit 1× dsDNA High Sensitivity kit (Invitrogen). Extracted DNA was sent for shotgun sequencing to the Australian Centre of Ecogenomics (ACE, University of Queensland). Sequencing was performed on the NextSeq500 platform using NextSeq 500/550 High Output v2 2x150bp paired-end chemistry with 5 Gbp sequencing coverage to yield at least 50 million paired-end reads per metagenome sample.

The quality of the reads was assessed using FastQC 0.11.4 (81). The paired-end reads were trimmed with Trimmomatic 0.39.1 using the paired-end mode to remove the Nextera adapters, leading and trailing N bases, leading and trailing bases below quality score 15, bases with an average quality score below 15 in every 4-base wide sliding window and reads below 35 bases in length (82). The quality paired-end reads were mapped to the ARS-UCD1.2 Bos taurus genome (GCA_002263795.2) (39) using Bowtie2 2.3.4.3 (83) to determine the number of cattle reads in each sample. SAMtools 1.3 (84) were applied to retrieve the alignments and BEDTools 2.25.0 (85) were used to extract paired-end reads of noncattle sequences. The microbial read percentage was calculated by getting the percentage of noncattle sequences in the total reads generated. The statistical details of the alignments were retrieved from the Binary Alignment Mapping (BAM) output files using SAMtools (84). The same procedure was used to remove human-read contamination by replacing the ARS-UCD1.2 Bos taurus genome with the GRCh38 Homo sapiens genome (GCA_000001405.15). The number of microbial and bovine reads was determined from the BAM output to compute the percentage of microbial reads.

### Read-based taxonomic classifications.

After quality was filtered and cleaned, the metagenomic reads were annotated taxonomically with Kraken2 (86). Kraken2 examined the *k*-mers information in the metagenomic reads and query the *k*-mers information against the database. The abundances, at the species level. of the organisms identified in the metagenomic samples, were computed using the Bayesian Reestimation of Abundance with KrakEN (Bracken) (87). Bioinformatic analysis and visualization were conducted on R studio (88) with R packages, including vegan 2.5.7 (89), phyloseq 1.34.0 (90), dplyr (91), and ggplot2 (92).

### Metagenomic assembly.

The quality paired-end reads of each sample were assembled into contigs using MEGAHIT 1.2.9 (93, 94), which incorporated *mercy-kmers* function to recover species with low abundances and were sequenced in low depth in a metagenome sample. The minimum contig length was limited to 200 bp. Additionally, the paired-end reads from all the samples were pooled to perform a coassembly using MEGAHIT 1.2.9. The reads were aligned back to the contigs using BBMap 38.84 (95) to determine the quality of the assemblies. The quality of the assembled contigs was also evaluated with MetaQUAST 5.0.2 (96). The coassembly was first analyzed using MetaQUAST against the SILVA 16S rRNA database (97) to identify the overall genome content of the metagenome samples. The identified genomes were downloaded from the NCBI database (98) to serve as the reference metagenome when evaluating the independent assemblies on MetaQUAST.

### Contig-based taxonomical and functional classifications.

The assembled contigs were annotated using an integrated pipeline MetaErg 1.2.0 (99). MetaErg performed HMMs profile similarity searches against several databases, including Pfam-A (100), TIGRFAM (101), FOAM (102), metabolic-hmms (103), and *cas*genes.hmm (104). MetaErg also performed DIAMOND (double index alignment of next-generation sequencing data) searches against Swiss-Prot (105) and the MetaErg in-built database GenomeDB. Mapping files generated from searches against Swiss-Prot, FOAM, and TIGRFAMs databases were incorporated in MinPath (106) to infer to KEGG (107) and MetaCyc (108) metabolic pathways. To weigh the relative abundances of the taxonomic, functional, and pathway compositions of the metagenome samples, individual BAM files were generated using BBMap (95) by aligning the metagenome reads of each sample to the coassembled contigs. A depth file was constructed with the jgi_summarize_bam_contig_depths script in MetaBat 2 (109) using the BAM files.

### Data availability.

The datasets generated during the current study are available in the NCBI sequence read archive (SRA) database under BioProject PRJNA786360 and BioSamples SRR17146641 to SRR17146676.
