# Supplementary material for: Evaluation of Host Depletion and Extraction Methods for Shotgun Metagenomic Analysis of Bovine Vaginal Samples
Source: Microbiol Spectr. 2022 Apr 11;10(2):e00412-21. doi: 10.1128/spectrum.00412-21 (PMC9045270; doi:10.1128/spectrum.00412-21)
Supplement: SUPPLEMENTAL FILE 1 — Supplemental material. Download spectrum.00412-21-s001.pdf, PDF file, 0.3 MB [file spectrum.00412-21-s001.pdf]

Supplementary Tables

Appendix 1: List of samples and whole-metagenome sequencing data details. The raw pair end reads were trimmed to remove the adapters and low-quality regions. Trimmed reads were mapped against the ARS-UCD1.2 *Bos Taurus* genome (GCA_002263795.2) to categorise the reads into cattle and microbial reads.

Sample type	Host-depletion method	Extraction method	Replicate	Giga base pairs (Gbps)	Raw paired end (bp)	Trimmed paired end (bp)	Cattle reads (bp)	Microbial reads (bp)
Vaginal swab	None	DNeasy	1	7.15	54,841,662	51,388,792	50,659,788	729,004
Vaginal swab	None	DNeasy	2	5.92	45,793,444	42,753,084	42,124,872	628,212
Vaginal swab+Spike	None	DNeasy	1	5.57	42,901,092	39,973,024	38,921,028	1,051,996
Vaginal swab+Spike	None	DNeasy	2	6.35	49,212,664	45,954,588	44,779,034	1,175,554
Spike only	None	DNeasy	1	6.03	45,667,332	43,195,152	166,720	43,028,432
Spike only	None	DNeasy	2	7.3	55,093,054	52,106,996	195,138	51,911,858
Vaginal swab	NEBNext	DNeasy	1	6.12	47,825,738	44,706,948	44,023,532	683,416
Vaginal swab	NEBNext	DNeasy	2	7	54,816,874	51,431,890	50,646,172	785,718
Vaginal swab+Spike	NEBNext	DNeasy	1	6.58	51,713,386	48,290,888	46,616,720	1,674,168
Vaginal swab+Spike	NEBNext	DNeasy	2	7.36	57,551,250	54,009,344	51,874,256	2,135,088
Spike only	NEBNext	DNeasy	1	7.93	61,163,078	57,815,130	185,122	57,630,008
Spike only	NEBNext	DNeasy	2	7.31	56,082,584	52,970,100	126,308	52,843,792
Vaginal swab	Soft-spin	DNeasy	1	8.65	66,896,442	62,943,414	62,073,970	869,444
Vaginal swab	Soft-spin	DNeasy	2	5.77	41,737,256	40,035,658	39,555,414	480,244
Vaginal swab+Spike	Soft-spin	DNeasy	1	5.8	42,922,514	41,035,622	40,055,080	980,542
Vaginal swab+Spike	Soft-spin	DNeasy	2	5.41	38,571,614	37,069,938	36,248,348	821,590
Spike only	Soft-spin	DNeasy	1	6.34	46,714,512	44,900,522	119,670	44,780,852
Spike only	Soft-spin	DNeasy	2	5.75	42,703,968	41,097,850	86,170	41,011,680
Vaginal swab	PMA	DNeasy	1	4.71	35,477,370	33,605,076	33,257,414	347,662
Vaginal swab	PMA	DNeasy	2	5.32	38,512,490	36,987,704	36,609,752	377,952
Vaginal swab+Spike	PMA	DNeasy	1	4.29	32,510,824	30,840,264	30,062,776	777,488
Vaginal swab+Spike	PMA	DNeasy	2	6.2	47,765,746	45,309,436	44,177,848	1,131,588
Spike only	PMA	DNeasy	1	5.66	41,158,146	39,606,368	605,142	39,001,226
Spike only	PMA	DNeasy	2	6.24	47,259,214	45,317,916	1,448,058	43,869,858
Vaginal swab	Soft-spin	QIAamp	1	8.04	82,197,126	75,261,330	69,884,668	5,376,662
Vaginal swab	Soft-spin	QIAamp	2	6	60,730,598	55,831,046	50,420,636	5,410,410
Vaginal swab+Spike	Soft-spin	QIAamp	1	9.24	87,737,012	81,253,078	61,515,620	19,737,458

Sample type	Host-depletion method	Extraction method	Replicate	Giga base pairs (Gbps)	Raw paired end (bp)	Trimmed paired end (bp)	Cattle reads (bp)	Microbial reads (bp)
Vaginal swab+Spike	Soft-spin	QIAamp	2	5.57	52,935,166	48,183,458	30,293,844	17,889,614
Spike only	Soft-spin	QIAamp	1	5.74	41,007,194	39,471,306	42,678	39,428,628
Spike only	Soft-spin	QIAamp	2	5.437	38,415,324	37,375,574	62,212	37,313,362
Vaginal swab	PMA	QIAamp	1	7.806	74,585,456	69,512,506	64,637,312	4,875,194
Vaginal swab	PMA	QIAamp	2	7.186	67,837,476	63,368,182	58,981,184	4,386,998
Vaginal swab+Spike	PMA	QIAamp	1	6.617	60,602,786	56,703,484	37,875,914	18,827,570
Vaginal swab+Spike	PMA	QIAamp	2	9.009	80,111,394	75,414,350	51,457,334	23,957,016
Spike only	PMA	QIAamp	1	5.509	38,463,722	37,318,694	134,698	37,183,996
Spike only	PMA	QIAamp	2	5.841	40,909,084	39,732,818	25,928	39,706,890

Appendix 2: Details of contigs generated for each sample, including the number of contigs, total bases, the length of the smallest and largest contig, mean contig length and minimum contig length needed to cover 50% of the genome (N50).

Sample type	Host-depletion method	Extraction method	Replicate	No. of contigs	Total bases (bp)	Smallest contig (bp)	Largest contig (bp)	Mean contig length	N50 (bp)
Vaginal swab	None	DNeasy	1	2,699	1,316,615	201	9,730	487	470
Vaginal swab	None	DNeasy	2	1,917	929,274	200	10,924	484	461
Vaginal swab+Spike	None	DNeasy	1	6,654	7,016,420	200	120,918	1,054	4,770
Vaginal swab+Spike	None	DNeasy	2	7,537	7,689,825	201	172,763	1,020	2,964
Spike only	None	DNeasy	1	598	16,386,754	200	541,271	27,402	141,929
Spike only	None	DNeasy	2	483	16,388,872	200	541,156	33,931	137,770
Vaginal swab	NEBNext	DNeasy	1	3,539	1,719,466	200	10,742	485	464
Vaginal swab	NEBNext	DNeasy	2	4,800	2,384,221	204	10,529	496	476
Vaginal swab+Spike	NEBNext	DNeasy	1	11,225	10,832,616	200	258,039	965	1,153
Vaginal swab+Spike	NEBNext	DNeasy	2	10,505	13,263,105	204	408,377	1,262	2,222
Spike only	NEBNext	DNeasy	1	426	16,376,645	200	541,271	38,442	134,863
Spike only	NEBNext	DNeasy	2	453	16,392,106	200	541,271	36,185	137,770
Vaginal swab	Soft-spin	DNeasy	1	3,158	1,536,980	200	11,016	486	472
Vaginal swab	Soft-spin	DNeasy	2	2,082	1,020,450	205	10,718	490	479
Vaginal swab+Spike	Soft-spin	DNeasy	1	7,526	8,005,646	200	258,924	1,063	1,890
Vaginal swab+Spike	Soft-spin	DNeasy	2	7,375	7,797,394	200	215,287	1,057	2,278
Spike only	Soft-spin	DNeasy	1	423	16,383,008	200	541,041	38,730	146,025
Spike only	Soft-spin	DNeasy	2	450	16,382,622	200	559,395	36,405	148,572
Vaginal swab	PMA	DNeasy	1	1,390	720,524	200	14,786	518	498
Vaginal swab	PMA	DNeasy	2	2,290	1,120,579	200	10,021	489	474
Vaginal swab+Spike	PMA	DNeasy	1	4,988	6,282,751	201	171,325	1,259	11,949
Vaginal swab+Spike	PMA	DNeasy	2	5,884	6,866,944	200	316,795	1,167	42,636
Spike only	PMA	DNeasy	1	364	16,401,196	200	1,427,014	45,058	195,667
Spike only	PMA	DNeasy	2	372	16,408,047	200	1,427,014	44,107	217,243
Vaginal swab	Soft-spin	QIAamp	1	19,711	23,362,645	200	764,995	1,185	37,221
Vaginal swab	Soft-spin	QIAamp	2	10,641	33,383,054	200	1,427,014	3,137	85,117
Vaginal swab+Spike	Soft-spin	QIAamp	1	364	16,401,196	200	1,427,014	45,058	195,667
Vaginal swab+Spike	Soft-spin	QIAamp	2	7,784	31,745,591	200	1,427,014	4,078	59,055

Sample type	Host-depletion method	Extraction method	Replicate	No. of contigs	Total bases (bp)	Smallest contig (bp)	Largest contig (bp)	Mean contig length	N50 (bp)
Spike only	Soft-spin	QIAamp	1	339	16,386,339	200	1,427,014	48,337	217,772
Spike only	Soft-spin	QIAamp	2	358	16,386,713	200	1,427,014	45,772	192,717
Vaginal swab	PMA	QIAamp	1	16,990	22,807,467	200	976,973	1,342	55,132
Vaginal swab	PMA	QIAamp	2	18,406	23,746,878	200	698,080	1,290	34,406
Vaginal swab+Spike	PMA	QIAamp	1	11,736	34,846,431	200	1,427,014	2,969	113,678
Vaginal swab+Spike	PMA	QIAamp	2	14,786	37,166,499	200	1,427,014	2,513	161,000
Spike only	PMA	QIAamp	1	362	16,411,379	200	1,427,014	45,335	218,956
Spike only	PMA	QIAamp	2	335	16,385,456	200	1,427,014	48,911	217,228
		co-assembly		54,409	94,842,349	200	1,426,252	1,743	16,111

Appendix 3: Evaluation on the quality of assembled contigs based on the by mapping the raw reads back to the assembled contigs.

Sample type	Host-depletion method	Extraction method	Replicate	Reads	Mapped reads	Mapped bases	Ref scaffolds	Ref bases	Percent mapped	Percent proper pairs	Average coverage	Average coverage with deletions	Standard deviation	Percent scaffolds with any coverage	Percent of reference bases covered
Vaginal swab	None	DNeasy	1	727,690	301,296	35,193,412	2,699	1,316,615	41.404	30.949	26.73	27.339	164.659	99.96	99.41
Vaginal swab	None	DNeasy	2	626,588	151,937	18,495,833	1,917	929,274	24.248	18.825	19.904	20.39	84.068	100	99.18
Vaginal swab+Spike	None	DNeasy	1	1,050,862	641,970	80,220,156	6,654	7,016,420	61.09	54.905	11.433	11.497	39.598	99.97	99.86
Vaginal swab+Spike	None	DNeasy	2	1,174,024	711,842	88,381,659	7,537	7,689,825	60.633	55.563	11.493	11.609	126.071	99.99	99.89
Spike only	None	DNeasy	1	43,025,676	42,862,980	5,551,458,965	598	16,386,754	99.622	99.217	338.777	338.788	421.022	100	99.99
Spike only	None	DNeasy	2	51,909,338	51,718,685	6,721,199,295	483	16,388,872	99.633	99.199	410.107	410.119	509.911	99.59	99.98
Vaginal swab	NEBNext	DNeasy	1	681,852	102,484	12,645,958	3,539	1,719,466	15.03	13.623	7.355	7.384	14.453	99.97	99.75
Vaginal swab	NEBNext	DNeasy	2	783,794	134,432	16,584,223	4,800	2,384,221	17.151	15.594	6.956	6.98	14.343	99.94	99.73
Vaginal swab+Spike	NEBNext	DNeasy	1	1,672,010	932,860	119,512,910	11,225	10,832,616	55.793	54.428	11.033	11.038	11.253	99.99	99.93
Vaginal swab+Spike	NEBNext	DNeasy	2	2,133,658	1,463,918	186,712,699	10,505	13,263,105	68.611	67.397	14.078	14.082	15.573	99.96	99.93
Spike only	NEBNext	DNeasy	1	57,623,102	57,412,871	7,308,581,788	426	16,376,645	99.635	99.247	446.281	446.293	528.067	100	99.99
Spike only	NEBNext	DNeasy	2	52,840,998	52,650,674	6,739,353,110	453	16,392,106	99.64	99.225	411.134	411.146	505.153	99.56	99.98
Vaginal swab	Soft-spin	DNeasy	1	867,496	265,576	30,454,244	3,158	1,536,980	30.614	23.927	19.814	20.186	268.28	99.87	99.46
Vaginal swab	Soft-spin	DNeasy	2	479,032	93,333	12,318,867	2,082	1,020,450	19.484	14.743	12.072	12.347	22.56	99.9	99.21
Vaginal swab+Spike	Soft-spin	DNeasy	1	979,344	641,789	84,282,428	7,526	8,005,646	65.533	58.524	10.528	10.615	86.163	99.95	99.85
Vaginal swab+Spike	Soft-spin	DNeasy	2	820,708	550,526	74,222,059	7,375	7,797,394	67.079	57.423	9.519	9.558	30.587	99.84	99.75
Spike only	Soft-spin	DNeasy	1	44,777,184	44,666,727	6,001,203,305	423	16,383,008	99.753	99.228	366.307	366.318	518.986	100	99.99
Spike only	Soft-spin	DNeasy	2	41,008,208	40,917,506	5,454,555,648	450	16,382,622	99.779	99.315	332.948	332.959	471.834	100	99.99
Vaginal swab	PMA	DNeasy	1	346,776	76,758	9,841,418	1,390	720,524	22.135	18.648	13.659	14.035	25.57	99.86	99.36
Vaginal swab	PMA	DNeasy	2	376,990	175,754	21,849,686	2,290	1,120,579	46.62	33.618	19.499	19.936	85.391	99.83	99.2

Sample type	Host-depletion method	Extraction method	Replicate	Reads	Mapped reads	Mapped bases	Ref scaffolds	Ref bases	Percent mapped	Percent proper pairs	Average coverage	Average coverage with deletions	Standard deviation	Percent scaffolds with any coverage	Percent of reference bases covered
Vaginal swab+Spike	PMA	DNeasy	1	775,510	517,559	68,858,469	4,988	6,282,751	66.738	62.904	10.96	11.033	48.01	99.94	99.89
Vaginal swab+Spike	PMA	DNeasy	2	1,130,310	726,188	96,029,299	5,884	6,866,944	64.247	61.426	13.984	14.044	82.712	99.95	99.87
Spike only	PMA	DNeasy	1	38,997,492	38,807,578	5,285,762,051	364	16,401,196	99.513	99.094	322.279	322.289	277.09	100	99.99
Spike only	PMA	DNeasy	2	43,864,144	43,646,582	5,728,632,021	372	16,408,047	99.504	99.076	349.136	349.147	317.065	99.73	99.99
Vaginal swab	Soft-spin	QIAamp	1	5,367,822	3,644,625	429,190,825	19,711	23,362,645	67.898	63.42	18.371	18.797	164.808	100	99.94
Vaginal swab	Soft-spin	QIAamp	2	5,392,182	3,921,852	498,673,309	24,630	28,712,852	72.732	69.164	17.368	17.573	109.121	99.99	99.93
Vaginal swab+Spike	Soft-spin	QIAamp	1	19,730,906	18,298,691	2,456,738,623	10,641	33,383,054	92.741	91.437	73.592	73.828	124.628	99.97	99.96
Vaginal swab+Spike	Soft-spin	QIAamp	2	17,885,164	16,635,633	2,348,322,720	7,784	31,745,591	93.014	92.146	73.973	74.054	124.734	99.92	99.97
Spike only	Soft-spin	QIAamp	1	39,426,638	39,336,402	5,427,855,513	339	16,386,339	99.771	99.194	331.243	331.252	234.489	100	99.99
Spike only	Soft-spin	QIAamp	2	37,309,692	37,236,319	5,226,620,514	358	16,386,713	99.803	99.279	318.955	318.965	242.56	100	100
Vaginal swab	PMA	QIAamp	1	4,870,746	3,756,915	475,904,447	16,990	22,807,467	77.132	72.134	20.866	21.167	108.542	99.99	99.93
Vaginal swab	PMA	QIAamp	2	4,380,988	3,221,566	413,918,096	18,406	23,746,878	73.535	68.512	17.43	17.662	99.054	99.99	99.93
Vaginal swab+Spike	PMA	QIAamp	1	18,818,460	17,819,195	2,478,913,505	11,736	34,846,431	94.69	93.894	71.138	71.201	168.28	99.98	99.97
Vaginal swab+Spike	PMA	QIAamp	2	23,946,990	22,949,097	3,196,014,333	14,786	37,166,499	95.833	94.825	85.992	86.111	176.887	99.97	99.97
Spike only	PMA	QIAamp	1	37,181,570	37,081,548	5,261,509,228	362	16,411,379	99.731	99.149	320.601	320.616	96.812	99.45	99.94
Spike only	PMA	QIAamp	2	39,640,874	39,476,422	5,595,809,155	335	16,385,456	99.585	99.023	341.511	341.53	172.327	99.4	99.99

Appendix 4: Evaluation on the quality of assembled contigs based on the mapped reads and coverage against a list of reference genomes detected in the samples.

Sample details				Genome statistics							Misassemblies		Unaligned				Mismatches	Statistics without reference						
Sample type	Host-depletion method	Extraction method	Replicate	Genome fraction (%)	NA50	NA75	LA50	LA75	Mapped (%)	Avg. coverage depth	# misassemblies	Misassembled contigs length	# fully unaligned contigs	Fully unaligned length	# partially unaligned contigs	Partially unaligned length	# mismatches	# contigs	Largest contig	Total length	N50	N75	L50	L75
Vaginal swab	None	DNeasy	1	0.034	...				49.91	28	7	3,362	2,201	1,105,333	0	0	2,040	2,699	9,730	1,316,615	470	372	894	1,685
Vaginal swab	None	DNeasy	2	0.016	...				44.7	24	4	1,747	1,666	830,250	0	0	954	1,917	10,924	929,274	461	366	616	1,186
Vaginal swab+Spike	None	DNeasy	1	0.934	1564	362	335	3453	67.11	11	105	594,434	2,140	1,144,040	72	208,286	41,389	6,654	120,918	7,016,420	4,770	533	208	2,169
Vaginal swab+Spike	None	DNeasy	2	1.028	1014	367	502	4012	68.47	12	104	959,845	2,277	1,227,551	72	230,916	44,643	7,537	172,763	7,689,825	2,964	524	226	2,649
Spike only	None	DNeasy	1	2.435	586619	167332	62	190	99.79	338	215	9,025,393	46	120,026	134	1,544,298	118,198	598	541,271	16,386,754	141,929	51,761	32	78
Spike only	None	DNeasy	2	2.435	613332	217111	62	170	99.8	410	218	9,159,124	60	135,241	130	1,534,325	117,978	483	541,156	16,388,872	137,770	62,512	33	76
Vaginal swab	NEBNext	DNeasy	1	0.084	...				24.06	9	16	7,752	2,374	1,195,246	4	3,485	4,917	3,539	10,742	1,719,466	464	374	1,207	2,248
Vaginal swab	NEBNext	DNeasy	2	0.138	...				26.3	8	28	18,874	2,986	1,512,855	6	4,104	8,073	4,800	10,529	2,384,221	476	379	1,603	3,016
Vaginal swab+Spike	NEBNext	DNeasy	1	1.405	875	342	1545	6680	61.01	11	141	1,752,890	3,504	1,986,212	69	321,886	58,423	11,225	258,039	10,832,616	1,153	552	982	4,526
Vaginal swab+Spike	NEBNext	DNeasy	2	1.714	1689	388	1175	5365	73.05	14	146	2,106,278	4,011	2,444,116	85	412,729	69,793	10,505	408,377	13,263,105	2,222	800	812	3,379
Spike only	NEBNext	DNeasy	1	2.436	613332	231160	62	168	99.81	446	226	9,178,760	42	67,616	125	1,592,375	118,052	426	541,271	16,376,645	134,863	62,380	34	77

Sample details				Genome statistics							Misassemblies		Unaligned				Mismatches	Statistics without reference						
Sample type	Host-depletion method	Extraction method	Replicate	Genome fraction (%)	NA50	NA75	LA50	LA75	Mapped (%)	Avg. coverage depth	# misassemblies	Misassembled contigs length	# fully unaligned contigs	Fully unaligned length	# partially unaligned contigs	Partially unaligned length	# mismatches	# contigs	Largest contig	Total length	N50	N75	L50	L75
Spike only	NEBNext	DNeasy	2	2.436	62357	24708	63	164	99.81	411	215	9,381,406	50	84,571	129	1,580,312	118,195	453	541,271	16,392,106	137,770	62,512	35	76
Vaginal swab	Soft-spin	DNeasy	1	0.043	...				49.79	25	6	3,050	2,550	1,273,347	0	0	2,461	3,158	11,016	1,536,980	472	373	1,053	1,980
Vaginal swab	Soft-spin	DNeasy	2	0.023	...				30.72	14	6	3,413	1,737	875,043	0	0	1,325	2,082	10,718	1,020,450	479	364	655	1,271
Vaginal swab+Spike	Soft-spin	DNeasy	1	1.08	10778	389	570	3867	71.23	10	117	811,615	2,247	1,188,395	70	277,588	45,060	7,526	258,924	8,005,646	1,890	581	253	2,602
Vaginal swab+Spike	Soft-spin	DNeasy	2	1.038	11113	373	560	3850	71.94	9	130	755,192	2,378	1,251,281	75	254,216	43,941	7,375	215,287	7,797,394	2,278	579	269	2,528
Spike only	Soft-spin	DNeasy	1	2.436	69806	25658	60	159	99.84	366	223	9,907,337	45	64,236	120	1,601,754	117,787	423	541,041	16,383,008	146,025	78,346	31	70
Spike only	Soft-spin	DNeasy	2	2.436	68539	22921	58	164	99.86	333	214	9,584,248	55	87,495	123	1,579,881	117,879	450	559,395	16,382,622	148,572	67,887	31	72
Vaginal swab	PMA	DNeasy	1	0.002	...				36.44	16	1	365	1,358	707,880	0	0	131	1,390	14,786	720,524	498	379	415	831
Vaginal swab	PMA	DNeasy	2	0.006	...				55.89	21	3	1,468	2,183	1,077,913	0	0	586	2,290	10,021	1,120,579	474	372	742	1,415
Vaginal swab+Spike	PMA	DNeasy	1	0.86	6824	401	160	2188	72.59	11	114	1,937,079	1,511	796,676	30	281,772	38,448	4,988	171,325	6,282,751	11,949	607	78	1,159
Vaginal swab+Spike	PMA	DNeasy	2	0.895	6830	342	90	2958	73.97	14	133	2,071,882	2,240	1,165,284	30	286,608	39,590	5,884	316,795	6,866,944	42,636	568	31	1,584
Spike only	PMA	DNeasy	1	2.436	799	326	54	132	99.63	322	230	11,149,723	80	87,205	99	1,594,917	117,987	364	1,427,014	16,401,196	195,667	108,590	23	50

Sample details					Genome statistics						Misassemblies		Unaligned				Mismatches	Statistics without reference						
**Sample type**	**Host-depletion method**	**Extraction method**	**Replicate**	**Genome fraction (%)**	**NA50**	**NA75**	**LA50**	**LA75**	**Mapped (%)**	**Avg. coverage depth**	**# misassemblies**	**Misassembled contigs length**	**# fully unaligned contigs**	**Fully unaligned length**	**# partially unaligned contigs**	**Partially unaligned length**	**# mismatches**	**# contigs**	**Largest contig**	**Total length**	**N50**	**N75**	**L50**	**L75**
					56	88																		
Spike only	PMA	DNeasy	2	2.436	80839	34266	55	132	99.62	349	223	11,146,903	83	87,892	101	1,603,635	117,986	372	1,427,014	16,408,047	217,243	107,007	22	50
Vaginal swab	Soft-spin	QIAamp	1	1.178	...	...	...	...	81.44	19	158	2,477,483	13,676	8,060,368	446	7,803,188	100,763	19,711	764,995	23,362,645	37,221	593	85	5,771
Vaginal swab	Soft-spin	QIAamp	2	1.387	...	...	...	...	81.5	17	179	2,902,998	17,231	11,333,369	887	8,567,382	138,031	24,630	790,541	28,712,852	1,795	654	840	8,209
Vaginal swab+Spike	Soft-spin	QIAamp	1	3.334	136774	307	...	...	96.13	74	288	11,141,846	7,175	5,401,547	359	7,678,007	168,785	10,641	1,427,014	33,383,054	85,117	17,304	85	312
Vaginal swab+Spike	Soft-spin	QIAamp	2	3.259	178862	260	...	...	95.7	74	264	10,716,692	5,303	6,265,840	361	5,683,970	153,703	7,784	1,427,014	31,745,591	59,055	14,386	99	390
Spike only	Soft-spin	QIAamp	1	2.437	81599	32688	53	131	99.87	331	226	11,244,282	38	61,473	100	1,601,980	118,039	339	1,427,014	16,386,339	217,772	108,590	21	48
Spike only	Soft-spin	QIAamp	2	2.436	77007	31442	57	139	99.88	319	224	10,847,048	51	66,154	101	1,601,631	118,014	358	1,427,014	16,386,713	192,717	94,617	25	55
Vaginal swab	PMA	QIAamp	1	1.141	...	...	...	...	87.3	21	146	2,181,627	12,054	7,550,792	564	8,038,473	102,357	16,990	976,973	22,807,467	55,132	708	56	4,284
Vaginal swab	PMA	QIAamp	2	1.236	...	...	...	...	85.2	18	151	2,899,165	12,121	7,879,456	572	8,037,192	108,531	18,406	698,080	23,746,878	34,406	686	66	5,031
Vaginal swab+Spike	PMA	QIAamp	1	3.416	14907	288	...	...	96.72	71	307	12,242,232	7,642	5,071,263	390	8,906,811	186,441	11,736	1,427,014	34,846,431	113,678	33,514	76	215

Sample details					Genome statistics						Misassemblies		Unaligned				Mismatches	Statistics without reference						
**Sample type**	**Host-depletion method**	**Extraction method**	**Replicate**	**Genome fraction (%)**	**NA50**	**NA75**	**LA50**	**LA75**	**Mapped (%)**	**Avg. coverage depth**	**# misassemblies**	**Misassembled contigs length**	**# fully unaligned contigs**	**Fully unaligned length**	**# partially unaligned contigs**	**Partially unaligned length**	**# mismatches**	**# contigs**	**Largest contig**	**Total length**	**N50**	**N75**	**L50**	**L75**
Vaginal swab+Spike	PMA	QIAamp	2	3.547	5967	313	...	...	97.34	86	353	13,283,730	9,615	5,932,366	525	9,514,056	213,234	14,786	1,427,014	37,166,499	161,000	25,345	63	186
Spike only	PMA	QIAamp	1	2.437	80839	32259	53	130	99.83	320	225	11,376,464	57	69,091	101	1,600,509	118,491	362	1,427,014	16,411,379	218,956	106,461	22	49
Spike only	PMA	QIAamp	2	2.437	80839	33663	54	132	99.7	341	222	11,227,761	38	66,565	100	1,597,713	117,930	335	1,427,014	16,385,456	217,228	107,007	22	49
